# Body-Worn IMU-Based Human Hip and Knee Kinematics Estimation during Treadmill Walking

**DOI:** 10.3390/s22072544

**Published:** 2022-03-26

**Authors:** Timothy McGrath, Leia Stirling

**Affiliations:** 1 Department of Aeronautics and Astronautics, Massachusetts Institute of Technology, 77 Massachusetts Avenue, Cambridge, MA 02139, USA; 2 Industrial and Operations Engineering, University of Michigan, 1205 Beal Avenue, Ann Arbor, MI 48109, USA; leias@umich.edu; 3 Robotics Institute, University of Michigan, 2505 Hayward St, Ann Arbor, MI 48109, USA

**Keywords:** IMU, human, joint angle, self-calibrating, biomechanics, knee, hip, soft tissue artifacts, treadmill, walking, gait

## Abstract

Traditionally, inertial measurement unit (IMU)-based human joint angle estimation techniques are evaluated for general human motion where human joints explore all of their degrees of freedom. Pure human walking, in contrast, limits the motion of human joints and may lead to unobservability conditions that confound magnetometer-free IMU-based methods. This work explores the unobservability conditions emergent during human walking and expands upon a previous IMU-based method for the human knee to also estimate human hip angles relative to an assumed vertical datum. The proposed method is evaluated (N=12) in a human subject study and compared against an optical motion capture system. Accuracy of human knee flexion/extension angle (7.87${}^{\circ }$ absolute root mean square error (RMSE)), hip flexion/extension angle (3.70${}^{\circ }$ relative RMSE), and hip abduction/adduction angle (4.56${}^{\circ }$ relative RMSE) during walking are similar to current state-of-the-art self-calibrating IMU methods that use magnetometers. Larger errors of hip internal/external rotation angle (6.27${}^{\circ }$ relative RMSE) are driven by IMU heading drift characteristic of magnetometer-free approaches and non-hinge kinematics of the hip during gait, amongst other error sources. One of these sources of error, soft tissue perturbations during gait, is explored further in the context of knee angle estimation and it was observed that the IMU method may overestimate the angle during stance and underestimate the angle during swing. The presented method and results provide a novel combination of observability considerations, heuristic correction methods, and validation techniques to magnetic-blind, kinematic-only IMU-based skeletal pose estimation during human tasks with degenerate kinematics (e.g., straight line walking).

## 1. Introduction

The quantification of human motion can support decision making related to human health and performance [1,2,3]. The typical gold standard technology to measure human motion is optical motion capture [4] (OMC). This method generally involves placing small reflective markers (typically reflecting in the infrared or visual spectrum) on the person and a calibrated array of optical receivers is then used to triangulate the location of these markers in space. To obtain joint angles or limb segment poses, the triangulated marker positions must be processed through a biomechanical modeling approach such as OpenSim [5] or the Vicon plugin gait model [6,7,8]. These mocap modeling approaches are often used as a “black box” and certain model limitations (e.g., assumptions of subject anthropometry or joint kinematics) may be not be apparent to every end user. Generally, OMC approaches have additional limitations, such as marker detachment or occlusion (i.e., camera line-of-sight requirement) and the requirement for a controlled, fixed capture volume in a laboratory [9].

An emergent technology to enable human motion measurement in the field is the use of inertial measurement units (IMUs) mounted to human limbs. These IMU packages typically consist of a gyroscope and accelerometer and are often coupled with a magnetometer. IMUs have a long tradition as navigation components in aerospace and nautical systems [10], which have led to a number of powerful algorithmic approaches to IMU orientation and position estimation. Researchers seek to couple well-known models of IMU sensor dynamics with novel human kinematic models in order to fuse human motion capture measures of interest from the body-worn IMU data. To date, IMUs have found use in many human motion applications, including clinical sciences [1,2,11,12,13], sports performance [14,15,16,17,18], activity recognition [19,20,21,22,23], occupational ergonomics [3], and even spacesuit fit evaluation [24].

When using IMUs to estimate human kinematics, it is important that knowledge of the alignment between the IMUs and the limb segments of interest is available. IMU-only kinematics methods can be classified into a few discrete categories [25]. Historical approaches have generally fallen in to two of these categories: *assumed alignment* and *functional alignment* approaches. In assumed alignment approaches, an IMU is attached to a body segment in such a way that its pose relative to the underlying limb anatomical frame is either approximately aligned or easily measured (e.g., [26,27,28,29,30]). In contrast, functional alignment methods first place IMUs on the body segments and then have the subject perform predefined motions or poses (e.g., [31,32,33,34]). These classes of approaches may employ varying levels of complexity in biomechanical modeling. While many of these methods have shown robustness and accuracy, there is a significant disadvantage: the requirement of specific alignment or calibration periods, which can be time-consuming or subject to errors when performed by a non-expert.

To address the limitations that arise from time-consuming IMU alignment and functional calibration, a new class of methods are being developed that require neither assumed alignments or predefined motions to estimate IMU-to-human body alignment: self-calibrating approaches. These approaches leverage kinematic models of how the human body moves under arbitrary motion to estimate relevant human motion measures. Researchers have considered one [35,36,37,38,39,40,41], two [42,43], and three [44,45,46,47] degree-of-freedom (DOF) joints (or combinations thereof). Among those self-calibrating methods that have considered 3-DOF rotational joints, the authors are aware of only one work (Adamowicz et al. [46]) that reports error of derived 3-DOF hip angles relative to an optical motion capture reference. Adamowicz et al. [46] used a novel Kalman filter-based approach to estimate relative orientation between adjacent sensors as well as relative orientation between sensor frames and their associated segment systems. This approach did use a magnetometer for full IMU orientation observability, in contrast to methods that avoid, minimize, or compensate the use of magnetometers (e.g., [37,40,44,47]) due to known limitations of magnetometers in time-varying or spatially heterogeneous magnetic fields [48]. Adamowicz et al. [46] reports hip angle root-mean-square-error (RMSE) during walking of 8.62${}^{\circ }$ in flexion/extension, 8.03${}^{\circ }$ in abduction/adduction, and 9.99${}^{\circ }$ in internal/external rotation relative to an optical motion capture reference.

An important consideration when developing self-calibrating IMU approaches to human motion estimation is the observability of constructed kinematic models, i.e., the ability of the model to infer its output from the measured IMU data. Self-calibrating approaches utilize kinematic models that are intended to describe more general human motion. Selection of the kinematic model, joint DOFs, and associated ranges of motion can influence observability. For example, consider modeling the human knee as a 1-DOF hinge joint. Exploiting the planar rotation of the knee hinge joint allows calculating relative heading between thigh- and shank-mounted IMUs when the knee’s hinge axis generally lies in the horizontal plane—a powerful advantage in magnetometer-free approaches (e.g., [47]). However, if applying general ball joint 3-DOF IMU-to-shared-joint-rotation-center models (e.g., [28,36,47]), a unique solution may not be attainable because the quasi-1 DOF knee joint cannot biomechanically explore the 3-DOF offered by the joint model. This condition would result in an unobservable kinematics model. Generally, this means there is no unique solution to the set of equations that define the kinematic model.

Few works in the IMU-based human motion estimation literature have considered observability. Salehi et al. [36] augmented the classical Seel et al. [35] method to preclude a latent unobservability condition that arose when angular velocity was similar between different IMUs on the lower body. Similarly, Nowka et al. [49] extended the Seel et al. [35] gyroscope-based hinge axis identification method and analyzed under which motion classes the axis identification equations yield a unique solution. Olsson et al. [41] extends previous work [40] to analyze local and global concepts of uncertainty in the joint axis identification. Their work allows understanding of if and when enough sufficiently-informative measurements have been taken to calibrate the system.

The current work offers three contributions the literature. First, the current work extends a previous self-calibrating lower-body kinematics estimator [47] (software released publicly as *bioslam* [50]) to address unobservability conditions that may arise during treadmill walking. Second, the current work extends this model to report hip angles in International Society of Biomechanics (ISB) convention. The newly-derived hip angles and knee angles reported from this method are compared to an optical motion capture reference. Third, this work provides a characterization of the effect of soft tissue artifacts on IMU-estimated knee angles during walking.

## 2. Model Identifiability

Following the terminology of Raue et al. [51], we differentiate between observability as an inherent property of a model and identifiability, i.e., the theoretical ability to determine unique parameter values from said model. The original model from McGrath and Stirling [47] included a short list of non-identifiabilities—two structural non-identifiabilities, i.e., non-identifiability of certain parameters due to inherent unobservability properties of the model and two practical non-identifiabilities, i.e., non-identifiabilities that are emergent due to insufficient quality or amount of the sensor observations. In support of the proposed extensions to the original model, a new practical non-identifiability is identified (No. 1 below) and a practical non-identifiability from the original paper is re-discussed in the specific context of hip kinematics modeling (No. 2 below).

(Practical non-identifiability #1) Non-identifiability of the four IMU-to-hip static joint center vectors when the measured human kinematic data does not sufficiently explore both hips’ DOFs. The hip is modeled as a 3-DOF free ball joint. Therefore the solution to the constrained joint center of rotation model is only unique and identifiable when each IMU flanking the joint rotates sufficiently in multiple DOFs relative to the joint center. This required multi-DOF rotation may not occur during walking if the hip kinematics are approximately 1-DOF (i.e., only flexion/extension). In the degenerate case of 1-DOF hip kinematics during upright walking the medial/lateral placement of the hip centers will become non-identifiable.
(Practical non-identifiability #2) Non-uniqueness of relative heading between sacrum and thigh IMUs. Without the use of magnetometer data, IMUs do not have an absolute heading reference. A kinematic model that introduces constraints between two IMUs (e.g., the constrained joint center of rotation model [47]) may discern a unique relative heading between the two IMUs, however, this condition is not guaranteed in general. In the aforementioned constrained joint center of rotation model [47], when one of the IMU-to-joint-center vectors is generally oriented normal to the heading plane (i.e., the thigh-to-hip-center vectors during upright walking), this non-uniqueness condition may become emergent. Note that the knee kinematics are different: the combination of (i) the hinge joint kinematic constraint and (ii) constrained joint center of rotation model is enough to provide an identifiable relative heading between the thigh and shank IMUs, even during upright walking.

## 3. Materials and Methods

### 3.1. Participants

Twelve total subjects (four male, eight female, age = 24.6 ± 3.0 years) participated in the study. The Committee on the Use of Humans as Experimental Subjects at MIT approved the study as protocol 1906898310. Exclusion criteria from participation in the study included (1) self-reporting or diagnosis of any lower extremity impairments that limit the subject’s walking ability; (2) self-reported or diagnosed health conditions that would prevent the subject for walking continuously for 30 min; (3) inability to walk on a treadmill for 30 min independent of an assistive device; and (4) lack of fluency in English.

### 3.2. Study Protocol

Each participant performed a simple functional calibration motion profile, detailed in McGrath and Stirling [47] (referred to as a “motion profile task” in [47]). Then, each participant walked for approximately 30 min on a treadmill at a self-selected walking speed (speed = 1.13 ± 0.18 m/s) in order to capture each subject’s natural treadmill gait. The calibration task was chosen to address practical non-identifiability (Section 2), and the walking task was chosen to mimic long-duration at-home monitoring opportunities—expanding beyond the short, episodic evaluations common in the clinic today.

**Figure 1:**
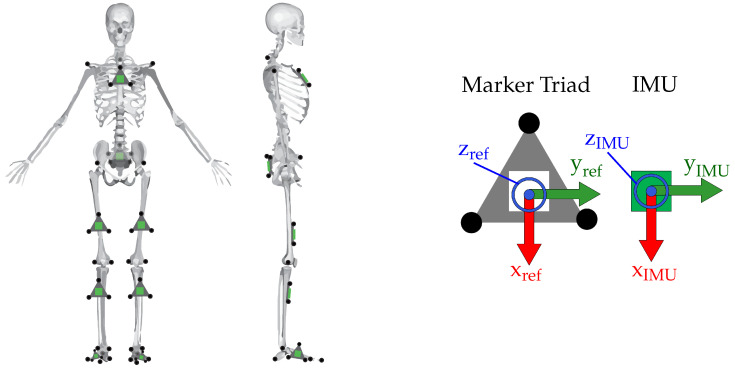
(**Left**) Placement of the reflective markers (black circles) and IMUs (green squares) on the subject. IMUs on the thigh and shank were not placed precisely, and location varied both vertically and in the transverse plane. (**Right**) A blown-up illustration of the marker triads with three markers affixed and IMU. Coordinate system of the IMU was known a priori, and the comparison reference coordinate system of the marker triad was constructed to match. Image from [47].

During treadmill walking, the subject was asked to reach a comfortable speed before data recording began, and data recording was ended approximately 30 min later before the subject ended walking. Motion capture markers and strap-on IMUs (Opal IMU, APDM, Inc., Portland, OR, USA) were placed on each subject (Figure 1). Optical motion capture markers were placed on anatomical landmarks according to the modified Cleveland Clinic lower body model marker set, commonly used in OpenSim [5,52] for inverse kinematics. Additionally, optical motion capture markers were mounted on each IMU via an acrylic plane that was affixed to the surface of each IMU. The position trajectory of all reflective motion capture markers was captured via a 13-camera Vicon motion capture system (Vicon Motion Systems, Inc., Los Angeles, CA, USA) sampled at 200 Hz. The IMUs contain dual 3-axis accelerometers (±16 g, ±200 g), and a 3-axis gyroscope (±2000 deg/s) sampled at 200 Hz. The recording of both IMU data and motion capture marker data started and stopped via a synchronized timing pulse, so that all measurements of IMU data and mocap marker data coincided.

### 3.3. Augmentation of the McGrath and Stirling Model for Pure Walking

For the pure walking task considered in this work (as opposed to the calibration motion profile considered in [47]), the specific limited kinematics during walking yield the two practical non-identifiability conditions detailed in Section 2. The McGrath and Stirling [47] model must therefore be adapted for use in a pure walking task.

The first addition to the original model is a prior model of the hip-connected joint center vectors from a calibration task. During pure walking, hip kinematics can become degenerate—not fully exciting all DOFs of the hip. These kinematics would manifest as the human performing primarily hip flexion/extension during gait (i.e., little internal/external rotation or abduction/adduction of the hip). When the kinematics are approximately this 1-DOF gait technique, the IMUs mounted on the thigh and lower back will not rotate enough relative to the hip joint center for the McGrath and Stirling [47] model to admit a unique solution for the four hip-connected joint center vectors. In the current treadmill walking task, this practical non-identifiability was shown to be emergent (practical non-identifiability No. 1 in Section 2). In order to address this practical non-identifiability, the estimated solution of the four hip-connected joint center vectors from the functional calibration task were used as model priors for the walking task.

An additional augmentation to the original model is the inclusion of a noisy hinge model for the ankle and hip joints. As described in Section 2 (practical non-identifiability No. 2), the heading relationship between adjacent IMUs is likely underspecified during pure walking for the IMUs, which flank the hip and ankle joints because the IMU to joint center vectors are generally oriented approximately normal to the heading plane. In order to preclude this non-identifiability condition for the 30 min walking trial, the hip and ankle joints were loosely modeled as kinematic hinges—the proximal and distal IMUs to these joints are constrained in the same way as the knee hinge in the original model [47]. The constraint is “loose” in the sense that non-hinge motion is permitted; however, as these joints have a tendency to act approximately as a hinge during upright gait, this constraint may enable model observability.

### 3.4. Derivation and Processing of Hip Angles

The McGrath and Stirling [47] model only derives information about the medial/lateral direction in the anatomical pelvic frame $L^{\prime }$ (i.e., from the vector spanning the hip rotation centers in the lumbar IMU frame L). An assumption about the pelvic proximal or anterior direction is necessary to construct a full definition of the pelvic coordinate system.

In the current study, the proximal direction in the pelvic frame, hereafter denoted as $v^{L^{\prime }}$, was assumed as the approximate average vertical (i.e., gravity) vector from the lumbar IMU. This assumption is only appropriate for upright gait (e.g., treadmill walking). Furthermore, some static offset biases between the IMU-derived hip angles and the OpenSim-estimated hip angles will be induced because the OpenSim gait 2392 model defines the pelvic distal/proximal direction as normal to the plane defined by the four anterior and posterior iliac spine markers.

An orthonormal pelvic coordinate system was constructed with the right direction (+x) fixed and the proximal direction (+z) corrected for orthonormality. The anterior direction (+y) was then derived from the cross product between *x* and *z*.

(1) $$R_{A}^{A^{\prime }}=f_{PCS}\left(x,z\right)=\left[\begin{array}{c} x^{⊤}\\ {\left(z\times x\right)}^{⊤}\\ {\left(x\times \left(z\times x\right)\right)}^{⊤} \end{array}\right]$$

Then, the orientation between the lumbar IMU and the pelvic coordinate system can be derived from $v^{L^{\prime }}$ and the normalized right direction vector,

(2) $$R_{L}^{L^{\prime }}=f_{PCS}\left(\frac{\left({\vec{s}}_{L}^{\;rh}-{\vec{s}}_{L}^{\;lh}\right)}{{∥{\vec{s}}_{L}^{\;rh}-{\vec{s}}_{L}^{\;lh}∥}_{2}},v^{L^{\prime }}\right)$$

where ${\vec{s}}_{L}^{\;rh}$ and ${\vec{s}}_{L}^{\;lh}$ denote the static vector from the lumbar IMU to the right and left hip joint centers, respectively. The hip’s three rotation angles are then reported according to Wu et al. [53]. (There is an inconsistency in segment coordinate system definition between Grood and Suntay [54] and Wu et al. [53], i.e., Grood and Suntay originally define the proximal direction of a segment to be +z but Wu et al. define it to be +x. In this work, +z is used for the proximal direction in all segments. The Wu joint coordinate system definition is then modified to report the same angles).

### 3.5. Data Processing

**Figure 2:**
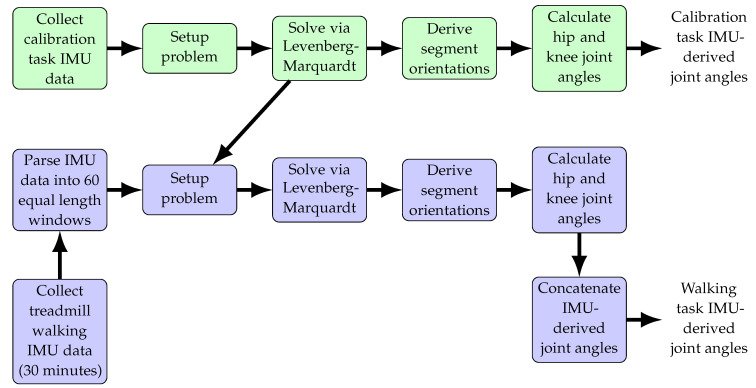
Conceptual process methodology to compute IMU-derived joint angles for both the calibration task (green) and the treadmill walking task (purple). Calibration task computed solutions the four hip-connected joint center vectors are used as priors in the walking task problem. Levenberg–Marquardt is used as an iterative solver to the proposed optimization problem. Knee joint angle results from the calibration task are reported in McGrath and Stirling [47]. Knee angles are derived according to Grood and Suntay [54] and hip angles are derived according to Wu et al. [53].

A methodological flowchart is shown in Figure 2 to illustrate the derivation of IMU-based joint angles for both the walking and calibration tasks. The estimation problem detailed in [47], augmented with the new constraints, was solved using Levenberg–Marquardt [55] within the GTSAM 4.0 library [56]. The 30 min dataset was cut into 60 equal-length subsets (approximately 30 s long each) to limit computational processing time and accommodate memory constraints. Criteria for convergence (identification of a local minima) included: an absolute change in error between iterations of $1\;\times \;10^{-6}$ or less, a relative change in error of $1\;\times \;10^{-4}$ or less, or 10,000 iterations, whichever was satisfied first. Then the heuristic identification and correction method detailed below was performed.

As a consequence of practical non-identifiability No. 2 in Section 2, hip internal/external rotation is generally non-identifiable. This may potentially lead to emergent local minima that trap the optimization routine and yield suboptimal solutions. A heuristic method was developed to detect drift of hip internal/external rotation angle (see Section 3.4 for derivation of the angle), correct it, and restart the optimization away from possible emergent local minima.

The method works through the following steps, once a minimum has been detected by the optimizer:

1. Compute both hip internal/external rotation angle sets.
2. Perform a best-fit simple linear regression on both hip internal/external rotation angle sets of form y=mx+b+e to identify slope *m* and intercept *b*, where *y* is the derived joint angle, *x* is time, and *e* is error.
3. If the absolute value of both hip internal/external rotation angle slopes *m* are less than some threshold $m^{*}$, this local minimum is determined to also be the global minimum. Exit. Otherwise, continue to step No. 4.
4. Set slope m=0 and compute new hip internal/external rotation angles $y^{\prime }$ such that *x*, *b*, and *e* are held constant.
5. Rotate the thigh, shank, and foot IMU poses such that the new hip internal/external rotation angles $y^{\prime }$ are induced.
6. Restart optimization from this new state.

In practice, this heuristic can be repeated indefinitely as long as objective function error continues to decrease. In the current study, this heuristic was repeated until converged solutions were less than 1% different than previous converged solutions.

The motion capture marker trajectory data were low-pass filtered with a 30 Hz, 6th order Butterworth filter. The data were then used to perform an inverse kinematics simulation in OpenSim 4.0 [5,52] using the gait 2392 model [57,58,59,60]. For each subject, a custom subject model was constructed by scaling the generic OpenSim gait 2392 model according to anthropometric measures derived from static marker data of the subject. All IMU data were processed according to the proposed model in [47] with augmentations described in Section 3.3. For each of the 12 walking trials, IMU-based time-series estimates of the hip and knee joint angles were compared to the OpenSim-estimated optical motion capture reference. Estimated knee angles are derived as detailed in [47] and hip angles are derived as detailed in Section 3.4. When comparing IMU-estimated joint angles to the motion capture reference, the IMU-estimated joint angle results from the equal-length subsets were concatenated to form a continuous dataset of IMU-estimated angles.

### 3.6. Data Analysis

The OpenSim-estimated joint angles (“mocap angles”) and the joint angles derived according to the proposed method (“IMU angles”) for the hip and knee angles were compared. There exists a static bias offset between the mocap and IMU angles. This bias may arise from the mocap marker placement or the proposed method. For example, a mocap bias emerges from small changes in the optical marker placement on the subject relative to the expected placement on the OpenSim model. The proposed model bias may emerge from the definition of the pelvic superior-pointing vector (Section 3.4), which is assumed from the approximate navigation vertical in the lumbar IMU frame. As a consequence, the proposed method has no absolute reference of pelvis orientation, leading to a similar lack of absolute datum for derived hip angles. Other factors that influence static offsets between the IMU and mocap angles are discussed in Section 4. To compare the mocap-derived joint angles to the IMU-derived joint angles, we define the relative IMU angle to refer to the adjusted IMU angle such that its mean is set to be the same as that of the mocap angle. Given the time-series IMU angle $\alpha_{1\ldots N}$ and mocap angle $\beta_{1\ldots N}$, the relative IMU angle $\alpha_{rel}$ would be computed as:

(3) $$\alpha_{rel,k}=\alpha_{k}+\frac{\sum_{i=1}^{N}\left(\beta_{i}-\alpha_{i}\right)}{N}\;\forall \;k=1\ldots N$$

The relative IMU angles are used to convey method error after subtracting datum offsets between the IMU and mocap data. Both the root mean square error (RMSE) and peak error of the difference between the estimated mocap angles and relative IMU angles are reported. In order to give additional context to the size of datum offsets between IMU angles and mocap angles, the absolute RMSE between IMU angles and mocap angles are also tabulated. Although the term RMSE is used, it should be noted that both signals are noisy estimates with inherent sources of error.

## 4. Results and Discussion

Absolute RMSE for both tasks is tabulated in Table 1. Table 2 and Table 3 respectively tabulate relative RMSE and relative peak error for both tasks. These results characterize method performance and explore emergent inter-subject variabilities for the proposed approach in a multi-subject pilot study; this study was not designed to test hypotheses comparing specific populations of participants or enable population-level inferences.

These results offer a few important takeaways. The proposed method estimates knee flexion/extension, hip flexion/extension and hip abduction/adduction to similar error as previous literature without the use of magnetometers to provide IMU heading, as evidenced by the error results reported in Table 1, Table 2 and Table 3. These error results suggest that the calibration task may yield lower error than the walking task. This result is expected, as the calibration task excites more DOFs of the human skeleton, yielding estimates of the model variables that more adequately capture the subjects’ kinematics. Considering the relative error data in context with absolute error highlights how model assumptions can affect derived joint angles. The relative RMSE supports a similarity in the time series of the data, while the absolute errors give context to the magnitude of bias sources that affect the estimates. For hip internal/external rotation specifically, absolute RMSE values have large mean and variance relative to the other skeletal DOFs, likely due to previously-discussed non-identifiabilities.

Average knee flexion/extension absolute/relative RMSE was found to be 7.87/3.77${}^{\circ }$ for the 30 min walking task and 4.30/2.77${}^{\circ }$ for the calibration task. These results are generally in line with the accuracy reported elsewhere in the literature, such as Seel et al. [35] who reported an average RMSE of 3.3${}^{\circ }$ for a single subject performing six gait tasks, or Cordillet et al. [61] who reported a mean knee flexion/extension RMSE between 3.74${}^{\circ }$ and 4.79${}^{\circ }$ during cycling, depending on choice of a priori calibration task. The work that inspired the kinematic model of the knee in the current method, McGrath et al. [38], reported a knee flexion/extension absolute/relative RMSE of 9.24${}^{\circ }$/3.49${}^{\circ }$ relative during gait. However, McGrath et al. [38] used simulated IMU data, which may underestimate method error through the removal of real-world sensor noises, whereas the proposed method results are reported for actual IMU data. McGrath et al. [38] also assumed the proximal IMU vector for the thigh and shank IMU, whereas the proposed method estimates these quantities from measured IMU data. The difference between absolute and relative RMSE can be driven by factors associated with both the IMU model and the optical motion capture model to which it was compared. The IMU model accuracy is affected by imperfect estimation of the static vectors from the IMUs to the joint centers. If these joint centers are improperly estimated to lie slightly anterior or posterior of their true values, a static offset in the derived joint angles would be induced. Additional sources of error may come from imperfect mocap data, such as mismatch between the placement of the physical mocap markers on the human subject and the virtual markers of the scaled OpenSim skeletal model. The OpenSim model is also imperfect, making assumptions about subject geometry and kinematic joint definitions. Soft tissue artifacts also induce error in all IMU-based methods, which is discussed in more detail later. Finally, all joint angles are estimated without a heading reference. It is reasonable to believe that if the subject were in an environment that allowed for robust magnetometer usage (such as outdoors)—and a well-calibrated magnetometer was available on the IMU package—that the estimated joint angles would have increased accuracy. This increased accuracy would arise because full orientation identifiability would be established rather than relying solely on the kinematics of the system (e.g., the hinge models and joint center constraint) to provide information on the heading relationship between IMUs.

It should be noted that only the flexion/extension angle is compared for the knee, as the mocap angles are based on the OpenSim gait 2392 model, which is a 1-DOF knee model. However, the proposed method also derives knee internal/external and adduction/abduction angles. Across all subjects, the (25th, 75th) percentiles of knee ROM during the walking task were (29.2${}^{\circ }$, 42.0${}^{\circ }$) for internal/external rotation and (21.1${}^{\circ }$, 26.3${}^{\circ }$) for abduction/adduction. In comparison, Deesloovere et al. [62] reports a knee internal/external rotation ROM of 14.41${}^{\circ }$± 4.1${}^{\circ }$ during walking. Lafortune et al. [63] reports a knee abduction/adduction ROM of 10${}^{\circ }$. Neither of these reported ROM values are within the (25th, 75th) percentile of the results from the proposed method. As the knee articulates, the motion of muscle and underlying tissue alters the relative orientation between the IMU and anatomical coordinate frames, which may yield the observed overestimation of the full ROM. Additionally, these observed ROM for the IMU-derived angles are possibly due to noises within the system (e.g. soft tissue artifacts) or suboptimal solutions from numerical optimization. Finally, the datasets analyzed in both the present study and general literature are taken from a small number of subjects and may not describe the true variability of ROM in the population at large.

Average hip flexion/extension angle relative RMSE was found to be 3.70${}^{\circ }$ and 7.15${}^{\circ }$ for the 30 min walking task and calibration task, respectively. Similarly, average hip abduction/adduction relative RMSE was found to be 4.56${}^{\circ }$ and 4.80${}^{\circ }$ for the two tasks. This accuracy is generally similar to other state-of-the-art IMU-based hip angle estimation techniques that incorporate magnetometers. For example, Adamowicz et al. [46] validates a novel filter-based approach that utilizes magnetic sensors to calculate hip angles. They reported a flexion/extension RMSE of 8.62${}^{\circ }$ for walking and 7.88${}^{\circ }$ for a chosen calibration task [64], and also abduction/adduction RMSE of 8.03${}^{\circ }$ in walking and 9.16${}^{\circ }$ in calibration. Chen et al. [65] developed a method that was also magnetometer-free, and reported a 2-DOF hip flexion/extension and abduction/adduction RMSE of 2.34${}^{\circ }$ and 2.95${}^{\circ }$, respectively, during gait; however, these results were only collected on ten seconds of gait data from a single participant. This experimental limitation may have precluded the Chen et al. method from observing time-varying phenomena, such as orientation drift or movement of the sensors on the human subject.

Accuracy of hip internal/external rotation angle in the current work was less robust. Relative RMSE was found to be 6.27${}^{\circ }$ and 8.18${}^{\circ }$ for the 30 min walking task and calibration task, respectively. However, the absolute RMSE gives additional context. For the walking task, hip internal/external rotation absolute RMSE was found to be 14.39${}^{\circ }$ with a large standard deviation of 7.81${}^{\circ }$. Adamowicz et al. [46] similarly saw a higher RMSE of I/E (9.99${}^{\circ }$± 5.90${}^{\circ }$) during walking than the other two hip DOFs. This large variance suggests notable and varied sources of bias in internal/external rotation angle estimation. Hip internal/external rotation angle estimation presents a low signal-to-noise ratio for IMU-based methods, in the sense that the signal (the hip internal/external rotation angle) is small in magnitude while the noise (namely, perturbation of the skin tissues) are prominent.

General hip angle estimation is confounded by the same error sources as discussed previously. One additional source of error in all hip angle estimation is the assumption of pelvic vertical (Section 3.4). Error between the assumed pelvic vertical from gravity and the true pelvic superior direction will induce error into the derived joint angles. A further possible source of error is the existence of poor hip hinge kinematics. If the hinge kinematic model is insufficient to provide information about relative heading angles between IMUs on the lower back and thighs, then it would be expected that hip internal/external rotation would not be well-estimated using this optimization framework. High error during walking may have been induced by non-hinge-like hip rotations during gait—a gait technique that likely varies by subject. Additionally, the actual calibration that was applied from the calibration task onto the 30 min walking task was only a prior on the four static vectors connected to the two hip centers. This calibration approach, while minimal, was likely insufficient to also provide practical identifiability of the heading relationship between lower back and thigh IMUs; it is possible that a calibration approach that also applies a prior on initial relative orientation between these IMUs or some prior on the actual internal/external rotation angle may remedy this problem. Alternatively, a “goodness” metric of hinge kinematics may be useful—a metric that informs when the hip is acting as a sufficient hinge system. This type of metric could allow for the hip to be selectively modeled as a hinge only during optimal periods. In the current work, a heuristic correction of hip internal/external rotation was developed to limit the drifting of hip internal/external rotation angles into unreasonable spaces (i.e., impossible or unlikely gait techniques). This heuristic enabled reasonable relative error values, but there is ample opportunity to further improve methods that handle this non-identifiability condition.

### 4.1. Soft Tissue Effects on Knee Angle Estimation

**Figure 3:**
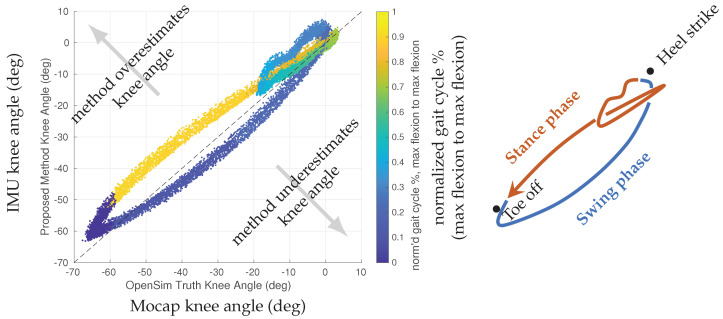
(**Left**) Subject 10 IMU vs. mocap left knee angle, colored by temporal location of the gait cycle. Gait cycle is defined to start and end at knee maximum flexion (approximately toe off) of the mocap angle. An ideal estimate would lie along the reference line with unity slope, shown as a black dotted line. Note the top-right corner of the data, where a disturbance between the heel strike and toe strike phase is observed. (**Right**) An approximate stylized representation of the gait cycle data, with swing (blue) and stance (orange) phases labeled, along with toe off and heel strike events.

Comparison between the estimated IMU and mocap angles also reveals information about the soft tissue artifacts impacting the system. Figure 3 shows the estimated knee angle plotted against the mocap-derived knee angle for an exemplary case during walking. If the knee angle estimate had no error, the data would lie along the line with slope equal to one. From the maximum flexion point of the knee, as the knee begins to extend (from toe-off through swing phase), the method tends to underestimate the knee angle value. As the knee reaches maximum extension (approximately heel strike, the top right of Figure 3), there is visible disruption due to soft tissue dynamics occurring at heel strike. This loop represents the loading response into mid-stance phase of gait. Through the rest of the stance phase, the IMU angle tends to overestimate the mocap knee angle.

In general, this plot is fairly elliptical except during the heel strike to beginning of foot flat region, where the most soft tissue perturbation is observed. The elliptical shape of this plot (overestimating the mocap angle in one phase vs. underestimating in another phase) is likely due to the tension and compression cycle of the skin tissue through gait. This shape may also be exacerbated by the contraction cycle of the muscles (e.g., quadriceps) during gait, fatty tissue perturbation, or possibly even assumptions of static knee hinge axis and static vectors from the IMUs to neighboring joint centers in the proposed IMU model. Additionally, this quasi-elliptical phenomenon may also be affected through factors of the optical motion capture model, such as static rotation axes or offsets between the virtually modeled markers vs. the actual placement of the markers on the subject. It should also be noted that the IMUs and motion capture markers likely experience different tissue dynamics. The IMUs are heavier and attached to the human via a thick elastic band. The motion capture markers are very light, attached only via a double-sided adhesive sticker. The mass of the IMU and tension of its elastic band may dampen higher-frequency perturbations that would otherwise affect the optical motion capture markers.

The general shape and perturbation effects of Figure 3 are generally seen across subjects. However, the magnitude of these effects, including the degree to which the IMU angle overestimates or underestimates the mocap angle, varies across subjects. These effects are likely driven by a complex combination of subject anatomy and anthropometry, such as height, fatty tissue presence, muscle tissue presence, as well as location of the IMU on the thigh and shank surface and subject gait technique. A larger subject study would be required to quantify the systemic factors that contribute to soft tissue perturbation.

This approach illustrates not only the existence of soft tissue artifacts between strap-down body-worn sensors and the optical motion capture model, but also suggests where in the gait cycle these perturbations are most prominent. Future work may include this approach in a larger analysis of the noise dynamics of the tissues of the leg on body-worn IMUs or use this approach to build heuristic correction models that are calibrated for specific subjects (i.e., outside of the heel strike to foot flat region, an elliptical correction of knee angle could be calibrated for a specific subject with only a few scalar parameters).

### 4.2. Future Work and Limitations

The current work used prior calibration of the four hip-connected static vectors to provide more accurate estimates during pure walking. The proposed method does not require a specific or precise calibration, any sufficient excitation of the hip DOFs will do. However, this may still present an operational limitation in scenarios where the hip is very constrained and cannot rotate in all DOFs. Future work can investigate additional kinematic descriptions of the hips to preclude the need for the specific calibration of these quantities. Additional advancements can be made through analyses that prescribe when sufficient calibration has been obtained, e.g., [41].

A further limitation in this work was the assumption of the vertical vector in the pelvic anatomical frame in Section 3.4. This assumption is only appropriate for generally-upright human poses (e.g., walking), but still injects a static bias into the estimated hip angles. These reported hip angles track the actual hip angle relative to a static bias, and can be useful for quantifying range of motion of the joint (among other uses). However, when being compared to an optical motion capture datum the relative (bias-removed) hip IMU angles had to be considered. Future work may seek to estimate the pelvic superior direction, which may lead to more robust hip angle estimation.

The proposed modeling framework may similarly derive ISB-convention 3-DOF ankle angles, however, these angles are not reported similarly by the OpenSim Gait 2392 model reference. This led to the ankle angles not being compared against a motion capture truth. Future work may select optical motion capture models that include the appropriate ankle models for comparison. This limitation also caused the two minor DOFs of the knee joint (internal/external rotation and abduction/adduction) to not be compared to an optical motion capture reference, although the range of motion was reported.

Future work may also consider examining the factors that influence the soft tissue characterization curve in Figure 3. With a larger dataset, it may be possible to construct a mixed-effects regression model that models the factors associated with a subject’s anthropometry and gait technique with a parameterization of the soft tissue characterization curve. Additionally, the current study did not examine older subjects or subjects with different body mass index. While the current study results show apparent soft tissue effects, these effects likely vary with subject age and body composition. The soft tissue effects, as well as the overall precision of the proposed method, should be further investigated for these individuals. Similarly, the subjects recruited for this study are not representative of the gender distribution of the population at-large, and this work, as experimentally tested, should not be used to infer gender-based differences in kinematics. Future work that extends this pilot study to consider more numerous and more representative subject selections may enable population-level inferences.

## 5. Conclusions

The current work expanded upon a previous magnetometer-free self-calibrating IMU-based joint angle estimation method to address an unobservability condition that commonly arises in pure human walking. Additions to the previous method include (1) utilizing a subject motion that explores all three hip DOFs (called a “calibration motion” in this work); (2) the prescription of a pseudo-hinge kinematics model to the hip in flexion/extension; and (3) a heuristic correction method of hip internal/external rotation angle. The heuristic correction method is easy to implement and based on a simple assumption of human walking kinematics. The proposed method was evaluated in an N=12 subject treadmill walking study where IMU-based 3-DOF hip angles were derived in ISB convention and compared against an optical motion capture system. Results suggest similar accuracy to comparable literature that uses magnetometers in hip flexion/extension and abduction/adduction estimation, however, hip internal/external rotation estimation showed larger error with large error variance. Additionally, it was found that the effects of soft tissue perturbation on knee angle accuracy may vary across the phases of gait, with an overestimation of knee angle during stance, an underestimation during swing, and a discrete perturbation due to impact at the point of heel strike. The approaches developed here advance IMU-based pose estimation techniques for operational use cases where walking tasks are considered in magnetically-heterogeneous environments.

## Figures and Tables

**Table 1:** RMSE (degrees) of absolute IMU joint angles vs. mocap joint angles for both the calibration and walking tasks. *F/E*, *I/E*, and *A/A* refers to flexion/extension, internal/external rotation, and abduction/adduction of the joint, respectively. Values are the average value for the right and left leg.

	Calibration Task				Walking Task			
Subject	Knee *F/E*	Hip *F/E*	Hip *I/E*	Hip *A/A*	Knee *F/E*	Hip *F/E*	Hip *I/E*	Hip *A/A*
**1**	3.10	10.09	13.90	6.06	4.42	4.07	15.06	15.97
**2**	4.54	9.40	20.62	6.00	5.80	3.70	13.09	10.28
**3**	6.77	9.75	18.65	4.59	12.06	8.00	32.44	12.07
**4**	4.63	8.21	15.47	5.22	9.03	4.28	9.68	15.20
**5**	4.24	5.28	15.55	9.37	12.35	15.12	27.08	17.31
**6**	4.93	5.31	16.33	7.11	4.22	18.64	6.97	15.14
**7**	4.77	11.88	13.75	5.63	8.30	7.63	10.92	10.11
**8**	4.23	10.40	15.23	9.81	12.75	10.25	6.57	15.92
**9**	5.32	11.62	9.28	4.49	8.53	3.84	9.43	11.25
**10**	3.22	7.09	15.46	7.41	4.64	12.22	15.69	17.43
**11**	3.38	8.46	10.47	5.44	8.22	9.10	13.08	17.67
**12**	2.42	5.32	8.86	4.15	4.14	4.92	12.61	13.64
**Mean**	**4.30**	**8.57**	**14.46**	**6.27**	**7.87**	**8.48**	**14.39**	**14.33**
**Std**	**1.16**	**2.38**	**3.54**	**1.83**	**3.27**	**4.86**	**7.81**	**2.79**

**Table 2:** RMSE (degrees) of relative IMU joint angles vs. mocap joint angles for both the calibration and walking tasks. *F/E*, *I/E*, and *A/A* refers to flexion/extension, internal/external rotation, and abduction/adduction of the joint, respectively. Values are the average value for the right and left leg.

	Calibration Task				Walking Task			
Subject	Knee *F/E*	Hip *F/E*	Hip *I/E*	Hip *A/A*	Knee *F/E*	Hip *F/E*	Hip *I/E*	Hip *A/A*
**1**	2.18	9.34	8.04	5.23	2.34	2.79	9.39	5.12
**2**	1.93	9.23	8.45	4.76	3.79	2.75	5.81	2.83
**3**	4.19	5.04	8.36	4.22	4.13	4.13	5.97	4.80
**4**	2.63	7.35	7.79	4.84	3.67	3.33	5.68	5.40
**5**	3.17	4.91	6.69	8.23	7.04	6.57	6.95	8.11
**6**	3.23	4.65	6.64	5.82	3.42	2.25	4.85	5.11
**7**	2.99	10.20	12.72	3.49	3.31	6.12	5.89	3.68
**8**	3.21	9.62	8.81	6.61	4.14	3.39	5.37	5.26
**9**	2.58	8.86	6.70	4.29	2.59	3.55	7.63	2.90
**10**	2.69	6.00	8.29	4.17	4.32	3.43	7.09	4.21
**11**	2.13	7.17	9.18	3.58	3.64	3.44	5.70	5.15
**12**	2.31	3.40	6.44	2.39	2.82	2.61	4.92	2.18
**Mean**	**2.77**	**7.15**	**8.18**	**4.80**	**3.77**	**3.70**	**6.27**	**4.56**
**Std**	**0.63**	**2.31**	**1.71**	**1.55**	**1.21**	**1.34**	**1.30**	**1.57**

**Table 3:** Peak error (degrees) of relative IMU joint angles vs. mocap joint angles for both the calibration and walking tasks. *F/E*, *I/E*, and *A/A* refers to flexion/extension, internal/external rotation, and abduction/adduction of the joint, respectively. Values are the average value for the right and left leg.

	Calibration Task				Walking Task			
Subject	Knee *F/E*	Hip *F/E*	Hip *I/E*	Hip *A/A*	Knee *F/E*	Hip *F/E*	Hip *I/E*	Hip *A/A*
**1**	7.80	40.22	24.21	23.64	9.73	12.44	38.55	13.78
**2**	8.28	46.25	26.19	15.60	22.65	17.13	23.43	19.40
**3**	16.56	23.64	38.71	12.63	14.94	14.13	25.03	14.55
**4**	9.50	33.33	26.53	16.15	10.33	11.87	27.52	13.46
**5**	15.04	19.13	28.09	34.49	18.79	18.45	26.49	34.02
**6**	17.42	23.27	24.38	15.45	10.83	9.65	18.77	12.33
**7**	10.07	51.20	34.84	12.80	9.10	39.82	29.79	10.50
**8**	16.51	58.11	38.12	19.83	12.06	13.12	24.88	13.72
**9**	12.30	41.89	24.59	11.79	7.70	18.72	28.96	10.39
**10**	10.44	38.79	28.72	20.03	11.46	13.73	36.07	13.38
**11**	8.12	43.60	31.96	14.56	12.02	14.22	27.28	13.84
**12**	9.84	14.17	24.60	8.71	8.78	7.30	19.99	7.16
**Mean**	**11.82**	**36.13**	**29.24**	**17.14**	**12.37**	**15.88**	**27.23**	**14.71**
**Std**	**3.60**	**13.57**	**5.37**	**6.81**	**4.41**	**8.24**	**5.76**	**6.74**

## Data Availability

The data presented in this study are available on request from the corresponding author.
